# News and Coming Events

**Published:** 1909-02-27

**Authors:** 


### News and Coming Events.

Her Majesty the Queen was present at the matinée given at the Queen's Theatre on February 18 in aid of the Royal Ear Hospital. The programme was exceptionally attractive and many prominent actors, actresses, and music-hall artistes contributed to the success of the entertainment.

---

The Board of Trade has received through the Foreign Office a gold watch awarded by the German Emperor to Dr. John Hargreaves Robinson, formerly surgeon of the British steamship *Avoca*, of Glasgow, in recognition of his services to the crew of the German steamship *Nordsee*, of Lübeck, when in distress at sea on May 17 last.

---

Mr. Alfred Temple Spanton died suddenly last week at Birkenhead at the age of thirty. He was educated at Cambridge and St. George's Hospital, and qualified in 1900. He was Medical Officer to the Great Central Railway, and had been Resident Medical Officer of the Leeds Public Dispensary.

---

The therapeutical and pharmacological section of the Royal Society of Medicine will meet next at 20 Hanover Square, W., on Tuesday, March 2, 1909, at 4.30 p.m., when a discussion on the Treatment of Spasmodic Asthma will be opened by Dr. Cecil Wall.

---

The annual meeting of the After-Care Association (whose object is to assist poor persons discharged from asylums for the insane) was held on February 3, at 26 Devonshire Place, W., Dr. G. H. Savage in the chair. After the reading of the Report a paper was read by Dr. Robert Jones, of Claybury, on "The Urgent Necessity of Helping Mental Convalescents."

---

Professor David James Hamilton, who recently resigned the Chair of Pathology at Aberdeen University, died at Aberdeen on February 19. He was the author of "The Pathology of Bronchitis" and many scientific and medical papers. He was educated at Edinburgh, Vienna, and Strassburg, and was formerly Demonstrator of Pathology at Edinburgh University. He was a member of the leading British pathological societies, and was a Fellow of the Royal Society

---

The annual Court of Governors of Charing Cross Hospital, held on February 18, marked the termination of the Earl of Kilmorey's period of chairmanship and the acceptance of office as his successor by Viscount Ridley. Lord Kilmorey has done admirable service towards redeeming the hospital from the desperate financial plight in which he found it three years ago, when he first took the chair. At that time the closing of the hospital was actually in contemplation, but now, mainly through his efforts, the state of affairs shows marked improvement: a debt of £16,000 has been paid off and the ordinary expenditure has been reduced from £20,000 to £18,322; while the hon. treasurer, Mr. W. R. Malcolm, is able to announce for the first time that there is no overdraft at the bank. The Earl of Kilmorey stated that the mortgage of £85,000 is now the principal burden that the institution has to carry. A sum of £1,000 has, however, been offered anonymously on condition that nine similar donations are made within two months. The outgoing Chairman expressed regret at the termination of the tenancy of the Orthopædic Hospital, and satisfaction at the fact that the King's Fund and the Hospital Sunday Fund year by year have increased their grants to Charing Cross Hospital.

The contributions received at the Hampstead General Hospital Festival Dinner on Tuesday evening, February 16, amounted, with a special donation of £1,500, to £4,575.

---

Sir William Collins, Vice-Chancellor of the University of London, was presented at the Guildhall on February 18 with the honorary freedom and livery of the Turners' Company in recognition of his high attainments in the art of surgery and medicine, and for his unwearied efforts in the cause of education and charity.

---

Dr. Charles Henry Felix Routh, M.D., M.R.C.P.(Lond.), died at Montagu Square, London, on February 19, at the age of eighty-seven. Dr. Routh, who was the eldest surviving son of Sir Randolph Routh, became a member of the Royal College of Surgeons in 1843, and subsequently a member of the Royal College of Physicians. He received his medical education at University College, London, Paris, and Vienna. He was consulting physician for diseases of women to the North London Hospital and to the Samaritan Hospital, and had held appointments at other medical institutions in the Metropolis. He was a corresponding Fellow of the Royal Academy of Madrid and the Academy of Pesth, and a member of the Society of Arts and of the Gynæcological Society of Boston, United States.

---

Owing to indisposition Lord Robert Cecil, K.C., M.P., was unfortunately prevented from presiding over the annual dinner of the London School of Clinical Medicine (Post-graduate) held at the Savoy Restaurant on February 19; and in his much-regretted absence the chair was taken by Mr. P. A. Nairne, Chairman of the Seamen's Hospital Society. The Chairman sketched the history of the Society, which when he joined it consisted of a ship on the Thames, and has since developed into an organisation having under its management two hospitals, two dispensaries, and two schools of medicine. Sir William Bennett endorsed the opinion of the Chairman and the Committee that the Clinical School is not a scheme intended for the benefit of medical men alone, but, like all post-graduate undertakings, is conducted for the good of the public; moreover, it is not intended to rival any other post-graduate institutions. Among those present were Viscount Ridley, Lord Lochee, Admiral Sir John Durnford, Sir W. Church (President of the Royal Society of Medicine), Sir Malcolm Morris, Sir H. Burdett, Sir Robert Burnet, Sir W. Bennett, Sir Thomas Smith, Sir W. Watson Cheyne, Sir W. J. Collins, M.P., Inspector-General J. Porter, Surgeon-General A. M. Branfoot, Canon Duckworth, Professor E. H. Starling, and Mr. H. W. Lucy.

---

The annual meeting of Queen Charlotte's Hospital was held on February 23, Mr. Frederick W. Hunt (Vice-President) in the chair. The report referred to the large increase in the number of in-patients (1,865, as compared with 1,701 in 1907) and of out-patients (2,169, against 1,996 in 1907), and to the very low maternal mortality. Amongst 4,165 out-patients during the past two years no death has occurred, while during the past 27 years 34,245 women had been attended in their own homes, with a maternal death-rate of slightly over one per thousand. There is a deficiency of £1,044 on the year's working, which, following on one of £673 in the previous year, causes the committee some anxiety. One hundred and thirty pupil midwives passed the Central Midwives Board examinations last year out of 139 who competed.

On Saturday, May 8, the Earl of Derby, the newly-installed Chancellor of the University of Liverpool, will confer the degree of Doctor of Laws, *honoris causa*, on Dr. Richard Caton, ex-Mayor of Liverpool, and Sir Donald Macalister, K.C.B., M.D., Principal of the University of Glasgow and President of the General Medical Council.

The first lecture of the course on National Eugenics was delivered by Professor Karl Pearson, F.R.S., on February 23, at the University of London, upon the subject of "The Purport of the Science of Eugenics." The subsequent lectures will be delivered by Mr. D. Heron and Miss E. Elderton.

The annual general meeting of the Royal London Ophthalmic Hospital (Moorfields Eye Hospital), City Road, E.C., will be held at the hospital on Friday, February 26, at three o'clock. Governors are invited to bring any friends who may be interested in the work of the hospital and would like to view the building.

Lord Sandhurst, Treasurer of St. Bartholomew's Hospital, has received from the Mercers' Company a further grant of £210 towards the Rebuilding Fund of the hospital; while the Committee for the removal of King's College Hospital to South London has received 150 guineas from the Mercers' Company (a second donation), bringing the total amount received from the Company up to £657 10s.

The Duke of Marlborough, K.G., President of the hospital, will preside at the annual Court of Governors of the Royal National Orthopædic Hospital, with which is incorporated the City Orthopædic Hospital, Great Portland Street, W., to be held on Thursday, March 4, at 4 p.m. The Court will be held in the Hall of the new Out-patient Department, in Bolsover Street, immediately behind the new hospital in great Portland Street.

The *London Gazette* for February 16 prints a notice, issued from the Privy Council Office, to the effect that a petition from the British Medical Association, praying for the grant of a Charter of Incorporation, has been presented to his Majesty in Council; and his Majesty having referred the petition to a Committee of the Lords of the Council, notice is further given that all petitions for or against such grant should be sent to the Privy Council Office on or before the 3rd day of April next.

